# Hypocretin-2 Saporin Lesions of the Ventrolateral Periaquaductal Gray (*vl*PAG) Increase REM Sleep in Hypocretin Knockout Mice

**DOI:** 10.1371/journal.pone.0006346

**Published:** 2009-07-22

**Authors:** Satvinder Kaur, Stephen Thankachan, Suraiya Begum, Meng Liu, Carlos Blanco-Centurion, Priyattam J. Shiromani

**Affiliations:** Veterans Affairs Boston Healthcare System, and Harvard Medical School, West Roxbury, Massachusetts, United States of America; Chiba University Center for Forensic Mental Health, Japan

## Abstract

Ten years ago the sleep disorder narcolepsy was linked to the neuropeptide hypocretin (HCRT), also known as orexin. This disorder is characterized by excessive day time sleepiness, inappropriate triggering of rapid-eye movement (REM) sleep and cataplexy, which is a sudden loss of muscle tone during waking. It is still not known how HCRT regulates REM sleep or muscle tone since HCRT neurons are localized only in the lateral hypothalamus while REM sleep and muscle atonia are generated from the brainstem. To identify a potential neuronal circuit, the neurotoxin hypocretin-2-saporin (HCRT2-SAP) was used to lesion neurons in the ventral lateral periaquaductal gray (*vl*PAG). The first experiment utilized hypocretin knock-out (HCRT-*ko*) mice with the expectation that deletion of both HCRT and its target neurons would exacerbate narcoleptic symptoms. Indeed, HCRT*-ko* mice (n = 8) given the neurotoxin HCRT2-SAP (16.5 ng/23nl/sec each side) in the *vl*PAG had levels of REM sleep and sleep fragmentation that were considerably higher compared to HCRT*-ko* given saline (+39%; n = 7) or wildtype mice (+177%; n = 9). However, cataplexy attacks did not increase, nor were levels of wake or non-REM sleep changed. Experiment 2 determined the effects in mice where HCRT was present but the downstream target neurons in the *vl*PAG were deleted by the neurotoxin. This experiment utilized an FVB-transgenic strain of mice where eGFP identifies GABA neurons. We verified this and also determined that eGFP neurons were immunopositive for the HCRT-2 receptor. *vl*PAG lesions in these mice increased REM sleep (+79% versus saline controls) and it was significantly correlated (r = 0.89) with loss of eGFP neurons. These results identify the *vl*PAG as one site that loses its inhibitory control over REM sleep, but does not cause cataplexy, as a result of hypocretin deficiency.

## Introduction

Neurons containing the neuropeptide hypocretin (HCRT), also known as orexin are located in the lateral hypothalamus and hypothesized to inhibit REM sleep [1], [2]. When these neurons are destroyed, either genetically [3]–[5] or through a chemical neurotoxin [6], REM sleep is enhanced. In humans, loss of HCRT neurons is associated with the sleep disorder narcolepsy and inappropriate triggering of REM sleep [1], [2]. There are two HCRT receptors (hypocretin-1 and 2 receptors) and in canine narcolepsy, the REM sleep abnormality results from a mutation in the HCRT-2 receptor [7]. Hypocretin-2 receptor knockout mice display narcoleptic behavior [8].

It is not known how HCRT regulates REM sleep. One possibility is that HCRT mediates its action via HCRT target neurons in the pons since REM sleep is generated from there [9], [10]. One hypothesized hypocretin target in the pons is the ventral sub lateral-dorsal region (vSLD), which is ventral to the locus coeruleus [11]. Lesions of HCRT receptor bearing neurons in this area in rats does increase REM sleep (56%) [12]. A second site is the ventral lateral periaquaductal gray (*vl*PAG) area where it is also hypothesized that neurons inhibitory to REM sleep are localized [13]. In cats, electrolytic lesions of the *vl*PAG [14] or microinjection of muscimol into the *vl*PAG [15] produces a long lasting increase in REM sleep. In rats, ibotenic acid induced nonspecific lesions of the *vl*PAG increases REM sleep [16].

The effects of lesions of this area on REM sleep have never been determined in mice. The benefits of using mice are that a murine model of narcolepsy exists and it provides a valuable tool to identify the circuitry underlying the disease. Moreover, transgenic mouse models exist where a reporter gene, such as green fluorescent protein (GFP), marks a specific phenotype of neurons and these could be used to ferret out the pharmacology of the *vl*PAG neurons. Therefore, the present study tests the hypothesis that *vl*PAG neurons are inhibitory to REM sleep by destroying these neurons and determining whether there is an increase in REM sleep. To destroy the *vl*PAG neurons a novel neurotoxin, hypocretin-2 conjugated to saporin (HCRT2-SAP), is used. The results reveal that *vl*PAG lesioned mice have a significant increase in REM sleep thereby identifying a hypocretin-to-*vl*PAG circuit that inhibits REM sleep.

## Methods

### Experiment 1: Effects of *vl*PAG lesions in HCRT-null mice

#### Animals

Mice (20–35 g) homozygous with respect to the deletion of the hypocretin gene on both alleles (HCRT*-ko* n = 18) were identified through PCR of tail snips. Heterozygote mice were not used as they do not display narcoleptic behavior [17]. HCRT-ko mice were obtained from Dr. Masashi Yanagisawa and Dr. Takeshi Sakurai and a breeding colony was established in our facility at the VA Boston Healthcare System in West Roxbury. The mice have been backcrossed for more than 20 generations on a C57BL/6J line and are congenic with respect to the C57BL/6J strain. Our recently published data from wildtype (C57BL/6J) mice (n = 9) were also used and provided a record of the normal sleep-wake pattern in this strain for comparison [18]. The WT mice were recorded at the same time and under the same conditions as the other mice in this study. The methods used to record and analyze the sleep data were the same for all mice. All animals were maintained on a 12∶12 h light-dark cycle with ad- libitum access to water and food. All experiments were conducted as per the guidelines of the American Association for the Accreditation of Laboratory Animal Care (AAALAC) and approved by the VA Boston Healthcare System's Institution Animal Care and Use Committee (IACUC).

#### Surgical procedures

Mice were implanted with sleep recording electrodes under isofluorane (4% for induction; 1.5% for maintenance) anesthesia as described previously [19]. Briefly, four jeweler's screws were inserted into the skull (two each atop the frontal and occipital cortex) and were used to record the electroencephalogram (EEG). Two flexible wires were inserted into the nuchal muscles and recorded the electromyogram (EMG). These electrodes were inserted into a plastic plug and secured to the skull with dental cement. The head of the mouse was adjusted on the nose bar so that bregma and lambda were at level height. At this time a single glass pipette (10–15 µm, tip diameter) was lowered into the *vl*PAG target area (relative to bregma)(anterior-posterior = −5.0; medial-lateral = ±0.8 mm; ventral to dura = −2.5 mm) [20] and either HCRT2-SAP (n = 11; 16.5 ng/23 nl/sec each side; Advanced Targeting Systems, San Diego, CA) or saline (n = 7; 0.9%, 23 nl/sec each side) were injected by a nanoliter pump (Nanoliter 2000, World Precision Instruments Inc., Sarasota, Fl). The glass pipette was left in place for a minute and then slowly withdrawn. Once outside the brain the patency of the pipette was confirmed by the appearance of a small droplet of either HCRT-SAP or saline at the tip of the pipette. HCRT2-SAP or saline were applied bilaterally to the *vl*PAG.

#### Recording of sleep and analysis of sleep data

After surgery all mice were housed singly in plastic cages with corn bedding. A week after surgery the mice were connected to flexible cables for recording sleep. Fifteen days after the injection the animal's sleep-wake patterns were recorded for 48 h.

The temperature in the sleep recording room was 22–24°C and a 12∶12 h light-dark cycle (7AM-7PM lights on; 100 lux) was maintained. Contralateral frontal-occipital EEG screw electrodes were used for EEG acquisition. The EEG data was filtered at 70 Hz (low pass filter) and 0.3 KHz (high pass filter) using a Grass polygraph and continuously sampled by the data acquisition program (Icelus software, developed by Mark Opp, University of Michigan, Ann Arbor, MI) at sampling rate of 128 Hz.

The EEG and EMG recordings were scored manually on a computer (Icelus software; Mark Opp, Univ Michigan, Ann Arbor, Michigan, USA) in 12 second epochs for wake, non-REM sleep and REM sleep by staff blind to the treatment. Wakefulness was identified by the presence of desynchronized EEG and high EMG activity. Non-REM sleep consisted of high amplitude slow waves together with a low EMG tone relative to waking. REM sleep was identified by the presence of regular EEG theta activity coupled with low EMG relative to slow wave sleep. After the EEG data were scored, the code was broken to reveal the identity of each mouse. The percent of time spent in wakefulness, non-REM and REM was determined for each hour. Two way-ANOVA followed by Holm-Sidak post-hoc test for multiple comparisons was used to compare changes in sleep parameters within the genotypes for the different treatment groups. Statistical significance was evaluated at the P<0.05 level.

The EEG and EMG recordings were also examined for signs of cataplexy, which is defined as a sudden loss of muscle tone during an awake episode. A previously described criteria was used for identification of cataplexy episodes [17], [18]. Cataplexy was identified if the mouse was awake for more than 2 minutes and then experienced a sudden loss of muscle tone. During these bouts, the EEG had diminished delta activity and predominant theta activity.

#### Immunohistochemistry to identify site of lesion

At the end of the experiment, the mice were euthanized (overdose of pentobarbital) and perfused transcardially with 20 ml phosphate-buffered saline at room temperature followed by 50 ml paraformaldehyde (4%) in phosphate buffer. The brains were removed and post fixed overnight in the same fixative and then transferred to 30% sucrose until it equilibrated. The brains were cut in a cryostat and 40 µ thick coronal sections obtained (1 in 4 series). The free floating sections were immunostained for NeuN (mouse anti-NeuN, MAB377, 1∶ 1K; Millipore, MA), followed by biotinylated-secondary antibodies. The secondary antibodies were visualized using ABC- DAB procedure. In HCRT*-ko* mice loss of NeuN staining demarcated the lesioned area and traced onto paper using a drawing tube attached to a microscope (Nikon Eclipse E400).

### Experiment 2: *vl*PAG lesions in FVB-GFP transgenic mice

Mice homozygous for the FVB-TgN (GadGFP) 45704Swn transgene, on a FVB background (FVB-GFP; n = 20; Jackson Laboratories, Bar Harbor, ME, USA) were used. These mice express enhanced green fluorescent protein (eGFP) under the control of the mouse Gad1 gene promoter, thereby revealing the GABA neurons [21].

These mice were implanted with sleep recording electrodes, and HCRT2-SAP (n = 12) or saline (n = 8) were microinjected into the *vl*PAG as described in experiment 1. The sleep recording procedure, and data analysis were same as in experiment 1.

At the end of the experiment all animals were euthanized (overdose of pentobarbital), perfused and the brains were removed. The brains were cut as mentioned above in experiment 1 and the free floating sections were immunostained for GFP (rabbit anti-GFP, 1∶ 20K; Millipore, MA) and NeuN (mouse anti-NeuN, MAB377, 1∶ 1K; Millipore, MA), followed by the respective biotinylated-secondary antibodies. The secondary reaction for the GFP was then visualized using the ABC-DAB and for visualizing the NeuN, we used ABC-AP Vector Red (SK-5100, Vector Labs, Burlingame, CA). Extent of lesion was marked by the boundary of the neuronal loss as seen by the NeuN staining and loss of the GFP neurons. The spread of the GFP neurons in the *vl*PAG area were plotted using a drawing tube and counted in both the lesion and control groups. GFP neurons were counted in every fourth section at three levels (AP levels−4.9 to 5.2) on both sides and averaged for each hemisphere.

Some of the FVB-GFP mice were injected with colchicine (3 mg/kg i.p.) and euthanized (overdose of pentobarbital) after 7 days (fixation as described in experiment 1). The sections from these brains were immunostained for localization of GABA (guineapig anti-GABA, NT-108 1∶1K; Protos Biotech Corp, NY), followed by the secondary conjugated to Alexa 568 (Goat anti Guineapig; Jackson Immunoresearch Laboratories, West grove, PA). Stained sections were then visualized and the images acquired using a confocal microscope (Nikon Eclipse TE 200-U microscope, Nikon Corporation, Melville, NY, USA) to identify the colocalization of GFP in GABA positive cells.

In tissue sections from the FVB mice stained with hypocretin antibody (goat anti-HCRT, sc8070, 1∶500, Santa Cruz Biotechnology, Santa Cruz, CA) we observed abundant HCRT fibers and terminals in the vicinity of the GFP positive neurons in the *vl*PAG area. Therefore, we also investigated if the HCRT-2 receptors are present on these neurons. Some sections from the FVB-GFP mice were treated with primary antibody to HCRT- receptor 2 (Goat anti-HCRT-R-2, sc 8074, 1∶50; Santa Cruz Biotechnology, Santa Cruz, CA) followed by the donkey anti goat secondary conjugated to Alexa-568 (Jackson Immunoresearch Laboratories, West grove, PA). The specificity of the HCRT-receptor 2 antibody has been demonstrated [22]. The images were then acquired and viewed using a confocal microscope.

### Experiment 3: Inputs to the *vl*PAG

To investigate the areas projecting to the *vl*PAG, the retrograde tracer, cholera toxin subunit b (23 nl; CTb 0.5%; List Biological Laboratories Inc.) was injected in the *vl*PAG in some of the mice (FVB-GFP; n = 8). One week later the mice were sacrificed (after overdose of pentobarbital), brains perfused, and tissue sections processed for immunohistochemistry (Goat anti CTb, 1∶30K, List Biological Laboratories Inc., CA) followed by the biotinylated secondary antibody, which was then visualized using the ABC-DAB procedure. Two of the wells from these injected mice were then also processed for the immunoreaction for the CTb (Goat anti CTb, 1∶2K, List Biological Laboratories Inc., CA), where CTb was visualized using secondary conjugated to alexa 568 (Jackson Immunoresearch Laboratories, West grove, PA). Following this reaction, one of these wells was then processed for a second reaction with hypocretin (rabbit anti HCRT sc8070, 1∶500, Santa Cruz Biotechnology, Santa Cruz, CA) and the other well for melanin concentrating hormone (Rabbit anti MCH, H-070-47, 1∶5K, Phoenix Pharmaceuticals Inc, Burlingame, CA). In these both hypocretin and MCH were visualized using the secondary antibody conjugated to the alexa 647 (Jackson Immunoresearch Laboratories, West grove, PA).

One of four series of sections was used to count the retrogradely labeled CTb cells on both sides of the hemisphere using a brightfield microscope at 10X magnification. The areas were identified based on established neural landmarks in mice [20]. Some of the areas with heaviest projections to the injection site involved counting two or three sections, which were then added to calculate the total number of projections to a targeted area.

## Results

### Experiment 1: Effects of *vl*PAG lesions in HCRT*-ko* mice

#### Extent of lesions in HCRT-ko mice

**Figure 1:**
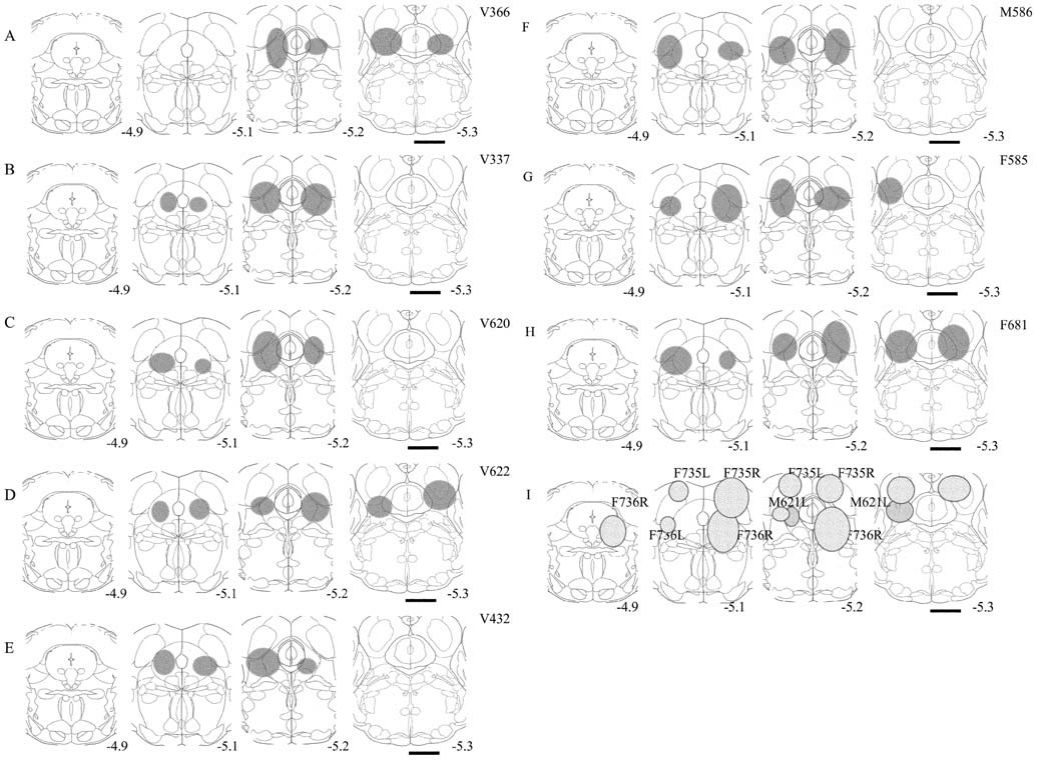
Schematic representation of the extent of lesions as defined by the boundary of loss of NeuN staining in the HCRT*-ko* mice. The lesions shown in A-H resulted in a significant increase in REM sleep, whereas in 3 mice (shown in I) the lesions did not significantly change REM sleep compared to the saline injected (no lesion) HCRT*-ko* mice. Scale bar = 1.0 mm.

**Figure 2:**
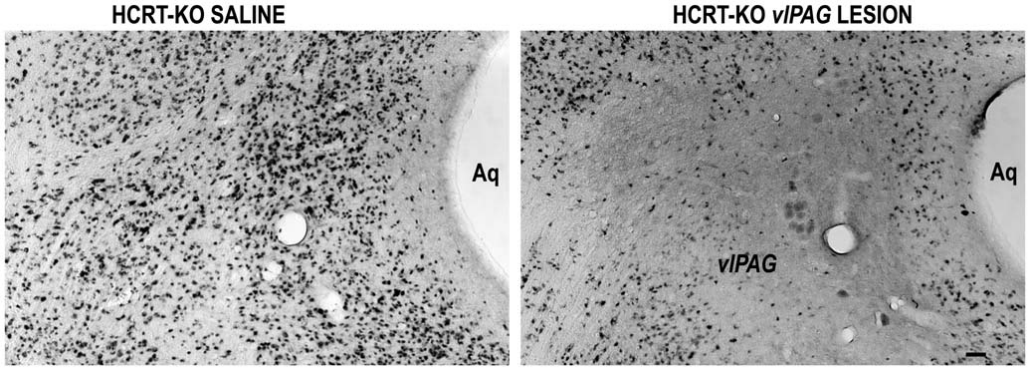
Photomicrographs of NeuN staining in the control (saline injected) and lesioned (HCRT2-SAP injected in the *vl*PAG) HCRT*-ko* mice. Compare with figure 9A which shows the localization of eGFP neurons in the *vl*PAG. Scale bar−50 µm.

In 8 of 11 mice HCRT2-SAP (16.5 ng/23 nl) induced lesions were localized to the *vl*PAG (fig. 1A-H). In the other three mice the lesions were off-target as follows: in one mouse the lesion was ventral (mouse F736); in another the lesion was dorsal to *vl*PAG (mouse F735); the third mouse had a unilateral lesion in *vl*PAG (mouse M621) (fig. 1-I). Figure 2 is a photomicrograph of lesion in the *vl*PAG as defined by loss of NeuN staining in a representative HCRT*-ko* mouse. In a separate group of eight WT mice (C57BL/6J), a microinjection of unconjugated saporin (16.5 ng/23 nl) was made to the vlPAG and observable neuronal loss such as that seen with the conjugated saporin (HCRT2-SAP)(figure 2) was not seen (Supplementary figure S1), which is consistent with other reports [23], [24].

#### Effect of HCRT2-SAP lesions of the vlPAG on sleep in HCRT-ko mice

**Figure 3:**
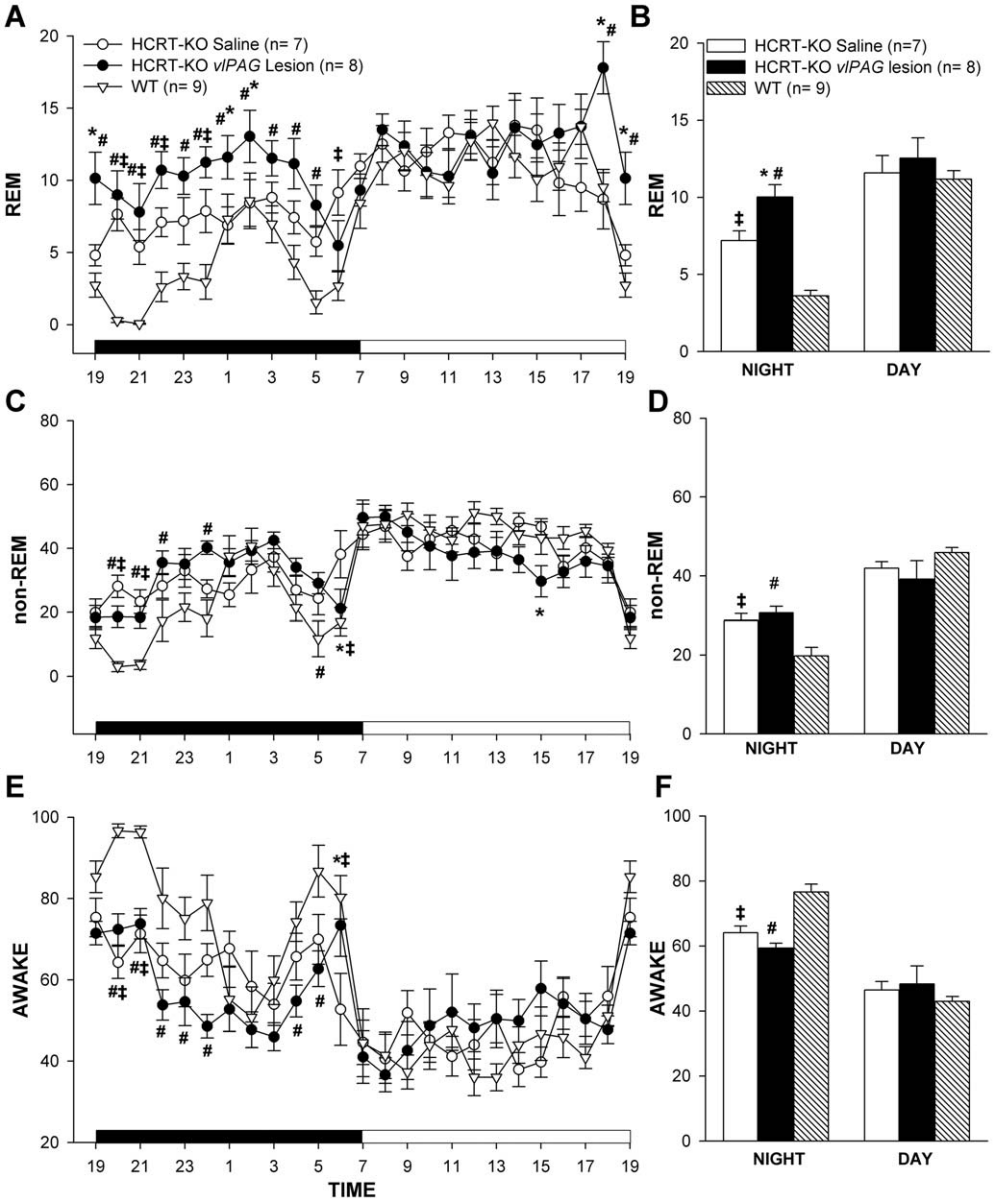
Mean (±SEM) percent REM, non-REM and wake states in HCRT*-ko* mice with *vl*PAG lesions compared to saline injected HCRT*-ko* and wildtype mice. The line graphs (A, C, E) summarize the hourly average of the sleep-wake states over the 24 h period. The dark bar (A, C, E) represents the lights-off period and the white bar represents the lights-on period of 12∶12 h of the L:D cycle. The bar graphs (B, D, F) represents the average (±SEM) REM, non-REM and wake levels during the 12 h day and night periods. * = P<0.01 for HCRT*-ko* saline vs. HCRT*-ko vlPAG* lesion, #- represents P<0.01 for WT vs. HCRT*-ko vlPAG* lesion and ‡- represents P<0.01 WT vs.HCRT*-ko* saline.

HCRT*-ko* mice with lesions of the *vl*PAG area (n = 8) had a significant increase in percentage of REM sleep (two-way ANOVA, for treatment×day/night, F_2, 42_ = 4.97, P = 0.012) during the night phase compared to saline in HCRT*-ko* mice (n = 7) or wildtype-C57BL/6J mice (n = 9) (fig. 3A and B). HCRT*-ko* mice already have more REM sleep at night compared to WT mice (99.63%), and the lesions increased it further (+177% versus WT; +39% versus HCRT*-ko*). Over the 24 h period REM sleep was increased by 20.34% compared to HCRT*-ko* mice given saline. The lesions had no significant effect on the percentage of non-REM and wake states compared to the HCRT*-ko* mice given saline (fig. 3C-F). There were no significant effects on REM, non-REM sleep and wake during the day between the two treatment groups of the HCRT*-ko* mice. The HCRT*-ko* mice (both saline and lesioned) had significant increase in non-REM (F_2, 42_ = 7.313, P = 0.002) and decrease in wake (F_2, 42_ = 8.245, P = <0.001) during the night phase compared to the wildtype- C57BL/6J mice (fig. 3C- F). Thus, *vl*PAG lesions in HCRT*-ko* mice exacerbated the increase in REM sleep but not the wake or non-REM sleep changes.

**Figure 4:**
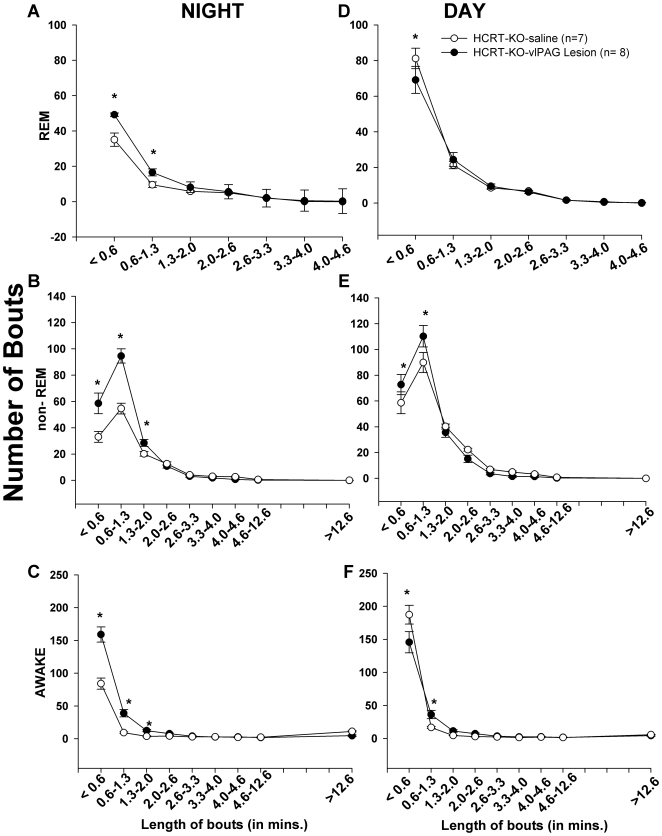
The number and length of bouts of awake, non-REM and REM sleep in saline vs. lesioned HCRT*-ko* mice. Each bout of awake, non-REM and REM sleep was placed in a bin of specific length (in minutes) of the bout. The figure represents the average (±SEM) number of bouts of a specific length in the lesioned and saline injected HCRT*-ko* mice. Asterisk (*) represents p<0.01 lesioned vs. saline HCRT*-ko* mice.

HCRT*-ko* mice have a more fragmented sleep-wake architecture compared to WT mice [17]. HCRT2-SAP lesions of the *vl*PAG in the HCRT*-ko* mice further destabilized the sleep architecture. Lesioned HCRT*-ko* mice had significantly more bouts of REM (F_1, 26_ = 5.99, P = 0.018), non-REM (F_1, 26_ = 5.109, P = 0.028) and wake (F_1, 26_ = 5.60, P = 0.022) during the night phase compared to the HCRT*-ko* mice injected with saline group (Table-1). This was a result of an increase in the number of short (<1.3 minutes) bouts of REM sleep (F_1, 13_ = 4.061, P<0.001), non-REM (F_1, 13_ = 15.017, P<0.001), and wake (F_1, 13_ = 6.328; p<0.001) compared to the saline injected HCRT*-ko* mice (fig. 4). In the lesioned mice, the average length of REM sleep bouts did not change at night compared to HCRT*-ko* mice given saline, but the average length of the non-REM (F_1, 26_ = 12.35, P = 0.002) and wake bouts (F_1, 26_ = 4.623, P = 0.041) significantly decreased during the night, which explains why the night time percentages of non-REM and wake were unchanged (Table-1). The increase in the number of bouts resulted in a significant increase (52.18 %) in the number of transitions during the night phase (F_1, 26_ = 5.860, P = 0.023) (Table-1).

**Table 1:** Effect of the HCRT-SAP induced lesions on the average (±SEM) number and length of bouts of wake, non-REM and REM sleep.

DAY/NIGHT	Group	number of REM bouts	length of REM bouts (min.)	number of NREM bouts	length of NREM bouts (min.)	number of Wake bouts	length of Wake bouts (min.)	Transition
**DAY**	HCRT-KO saline (n = 7)	10.02±0.78	0.75±0.031	19.31±1.55	1.42±0.12	19.39±1.56	1.43±0.13	583.71±46.63
	HCRT-KO *vl*PAG lesion (n = 8)	9.32±1.10	0.83±0.056	20.30±1.33	1.11±0.11 ‡	20.33±1.34	1.61±0.31	598.44±42.01
	FVB-GFP saline (n = 8)	4.95± 0.86	1.01± 0.12	12.26± 2.44	4.17± 1.29	12.31± 2.45	2.96± 0.62	354.13± 66.99
	FVB-GFP *vl*PAG lesion (n = 8)	5.96± 1.56	0.87± 0.15	12.45± 2.61	3.77± 1.43	12.53± 2.61	4.24± 1.27	371.32± 80.33
**NIGHT**	HCRT- KO saline (n = 7)	4.82±0.32	0.88±0.03	11.21±0.78	1.45±0.08	11.32±0.80	3.79±0.43	314.71±26.81
	HCRT- KO *vl*PAG lesion (n = 8)	6.93±0.62 ‡	0.88±0.08	16.42±1.06 ‡	1.09±0.07 ‡	16.65±1.05 ‡	2.34±0.25 ‡	478.88±28.26 ‡
	FVB-GFP saline (n = 8)	3.03± 0.42	1.24 ±0.09	7.75±1.31	4.11±1.07	7.79±1.33	5.10± 0.67	222.75±35.90
	FVB-GFP *vl*PAG lesion (n = 8)	6.19±1.05*	1.20±0.17	11.58±2.03	3.32±1.04	11.65±2.04	3.01±0.53 *	353.00±60.56

Sleep transitions represent the tally of entries into wake, NREM and REM sleep.

* FVB-GFP *vl*PAG lesion vs. FVB-GFP saline; P<0.01.

‡ HCRT-KO saline vs. HCRT-KO-*vl*PAG lesion; P<0.05.

#### Effects on cataplexy

**Figure 5:**
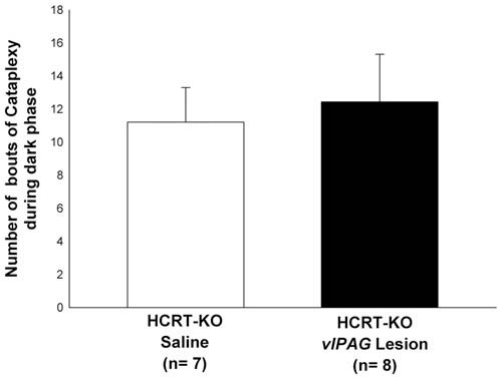
Mean (± SEM) number of cataplexy bouts during the 12 h lights-off period in lesioned and non-lesioned (saline) HCRT-*ko* mice.

The number of cataplexy bouts did not change in HCRT*-ko vl*PAG lesioned mice compared to HCRT*-ko* mice given saline (fig. 5). In the HCRT*-ko* mice cataplexy is distinct from REM sleep [25] and although the number of REM sleep bouts increased the incidence of cataplexy did not.

### Experiment 2: Effects of *vl*PAG lesions in FVB-GFP transgenic mice

#### Extent of lesions in FVB-GFP mice

**Figure 6:**
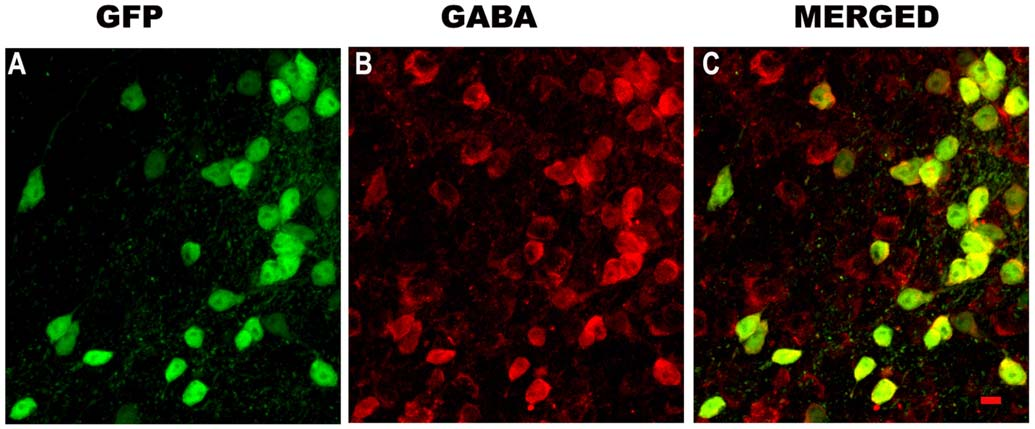
Neurons in the *vl*PAG that express green fluorescent protein (GFP) are also GABA positive. FVB-TgN(GadGFP)45704Swn transgenic mice express GFP under the control of the promoter for the gene encoding the GABA synthesizing enzyme, glutamate decarboxylase-1 (GAD-1; synonymous with GAD-67). To verify that GABA is indeed present in GFP neurons, tissue sections containing the *vl*PAG from these mice were processed for immunohistochemical detection of GABA. The tissue sections were mounted onto slides and the images were obtained using a confocal laser microscope under 60×magnification. The merged image indicates that every GFP neuron also contains GABA (Yellow). Some GABA positive neurons were not GFP positive, but this may be due to the particular type of GAD promoter used to express GFP. Scale = 10 µm.

**Figure 7:**
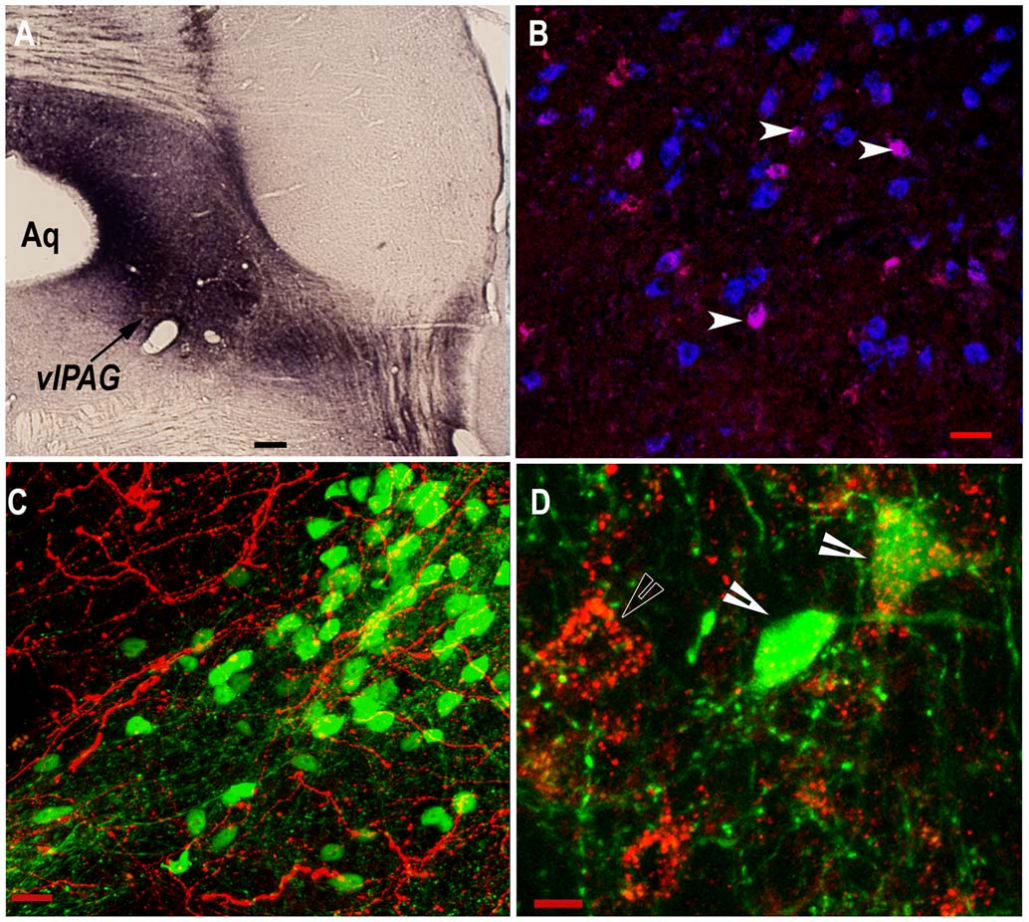
Hypocretin innervation of the *vl*PAG. The retrograde tracer, CTb, was microinjected to the *vl*PAG (shown in A). In photo B, HCRT immunoreactive neurons are identified in blue and some of these were also positive for the retrograde tracer (arrowheads in photo B) indicating projection of HCRT neurons to the *vl*PAG. Photo C indicates that the *vl*PAG area where the GFP neurons (green) are located is traversed by hypocretin fibers and terminals (red). The image in D shows the presence of hypocretin-2 receptor immunoreactivity (red) in the GFP neurons (as shown by the filled arrowheads) in the *vl*PAG area. The unfilled arrowhead in D shows the presence of HCRT-2 receptor immunoreactivity in non-GFP labeled neurons. Scale in A = 100 µm, B = 25 µm, C = 10 µm, D = 5 µm.

In the FVB-GFP mice the GFP neurons are distributed along the medial-lateral and rostral-caudal extent of the *vl*PAG. First, we verified that virtually all of the GFP labeled neurons in the *vl*PAG were also GABA immunoreactive (fig. 6). Next, we determined that the *vl*PAG was innervated by HCRT neurons. Injection of CTb, a retrograde tracer, in the *vl*PAG resulted in retrogradely labeled somata in the lateral hypothalamus, and some of these were HCRT neurons (fig. 7A and B). We also observed abundant hypocretin terminals in the vicinity of the GFP neurons in the *vl*PAG (fig. 7C). Most of the GFP neurons were immunoreactive for the hypocretin-2 receptor (fig. 7D). The specificity of the antibody for the hypocretin-2 receptor has been verified [22]. To confirm in mice, we examined another region such as the locus coeruleus where the hypocretin-1 receptor is present [26], [27], and did not find hypocretin-2 receptor immunoreactive neurons.

**Figure 8:**
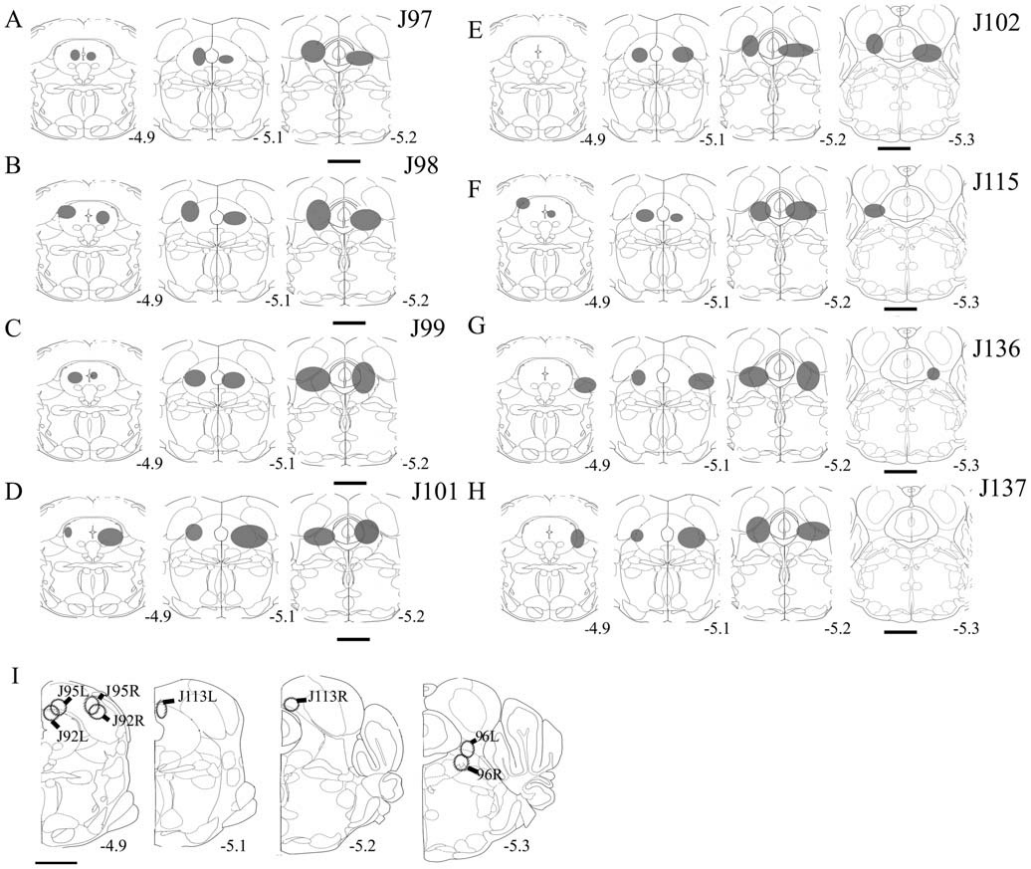
Schematic representation of the extent of lesions as defined by the absence of NeuN staining and the loss of GFP neurons in the *vl*PAG area of the pons in the FVB-GFP mice. The lesion extent shown in the pontine sections of the mice in A-H, significantly increased REM sleep during the dark phase, but the lesions in some mice (n = 4, the tip of the injections shown in I) did not cause significant changes in night time REM sleep compared to the saline injected control mice. Scale−1.0 mm.

**Figure 9:**
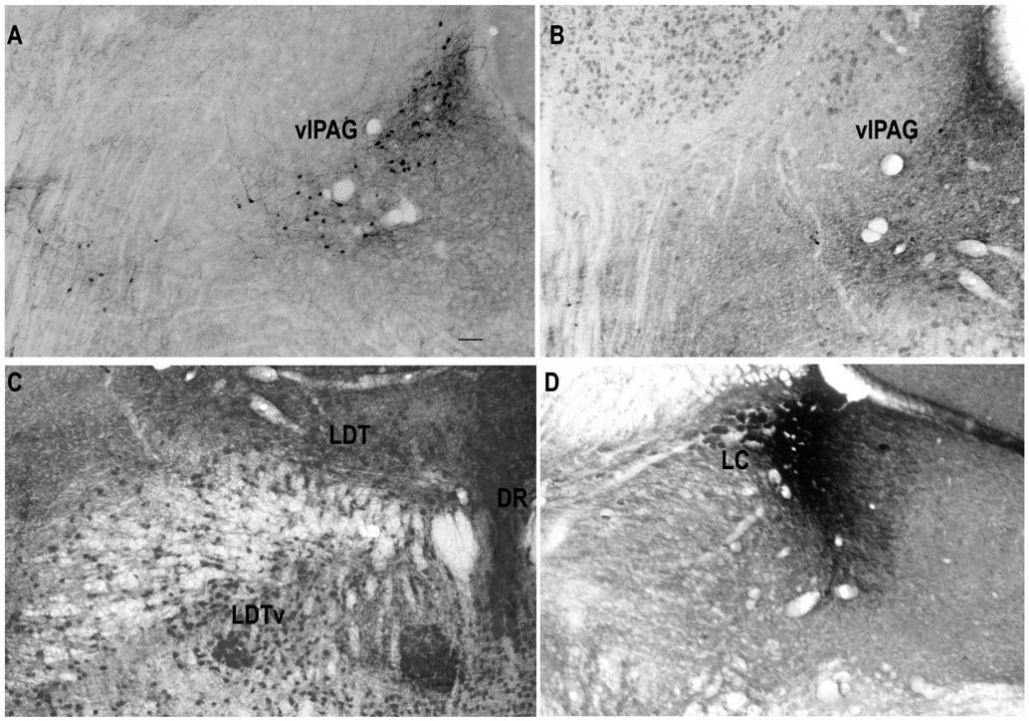
Photomicrographs showing the loss of the GFP neurons in the control (A) and lesioned FVB-GFP mouse (B). The cells in the lateral dorsal tegmental area (LDT) and medial dorsal raphae (DR) are spared by the lesions as shown by the NeuN staining (C), Similarly the noradrenergic neurons of the locus coeruleus (D) are also spared by the lesion. Scale−50 µm.

Since the GFP neurons were also GABA positive, these were counted and compared between the lesioned and the unlesioned group. However, the extent of the lesioned area was also determined by NeuN staining. There were an average of 63.46±4.81 (n = 7) GFP neurons per hemisphere (AP levels−4.9 to 5.2) in FVB-GFP mice given saline. Since we counted one out of every four sections, we estimate that there are about 252 GFP neurons in the vlPAG per hemisphere in FVB-GFP mice. Microinjection of HCRT2-SAP (16.5 ng/23 nl) in eight of the injected FVB-GFP mice (fig. 8A-H) resulted in an average loss of 74.41%±5.17 (ranged from 55–92%; fig. 8A-H) of GFP neurons, while in four of the mice (fig. 8I) only 7–20% of the GFP cells were lost. These four mice were excluded from the sleep-wake data analysis. In lesioned FVB-GFP mice (n = 8) the number of GFP neurons in the *vl*PAG was significantly low (two way ANOVA, for treatment×levels, F_1, 42_ = 20.27, P<0.001) compared to saline FVB-GFP mice (n = 8). Figure 9 depicts the extent of the HCRT2-SAP induced lesions in the FVB-GFP mice. Figure 9A is a photomicrograph of the GFP labeled neurons in the *vl*PAG area of FVB-GFP mice given saline. Figure 9B is the *vl*PAG area from a representative FVB-GFP mouse given the neurotoxin and shows the loss of the GFP neurons along with NeuN neurons. Lesions did not encroach onto areas adjacent to the *vl*PAG such as the laterodorsal tegmentum, dorsal raphe or the locus coeruleus (fig. 9C and D).

### Effect of HCRT2-SAP lesions of the vlPAG on sleep in FVB-GFP mice

**Figure 10:**
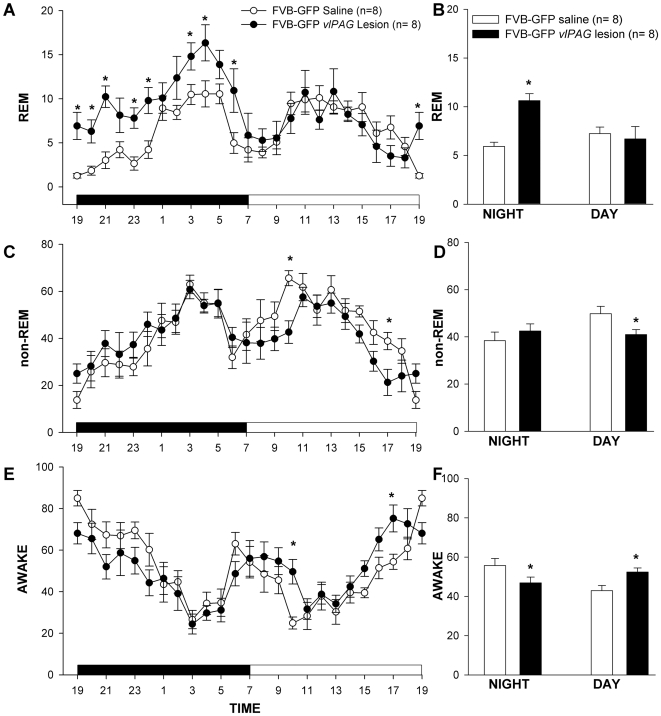
Mean (± SEM) percent REM, non-REM and awake states in FVB-GFP mice with no lesions (saline injected) and with HCRT2-SAP *vl*PAG lesions. The line graphs (A, C, E) summarize the hourly average of the sleep-wake states over the 24 h period. The dark bar (A, C, E) represents the lights-off period and the white bar represents the lights-on period of 12∶12 h of the L:D cycle. The bar graphs (B, D, F) represents the average (± SEM) REM, NREM and wake levels during the 12 h day and night periods. Significance levels- single asterisk (*) represents the P<0.01 for FVB-GFP saline vs. FVB-GFP *vlPAG* lesion.

FVB-GFP mice with lesion of the *vl*PAG area (n = 8) had significantly more (79.09%) REM sleep (F_1, 28_ = 9.85, P = 0.004) during the night compared to FVB-GFP mice given saline (n = 8). There was no increase during the light phase. Indeed, the lesions caused a reversal in the day-night distribution of REM sleep so that night-time REM was higher than daytime (Fig. 10B). Thus, the diurnal rhythm of REM sleep was changed in that REM sleep levels at night were higher compared to day in lesioned mice. As a result of the night time increase in REM sleep, there was a 43.4% increase in REM sleep over the 24 h period.

There are differences in sleep, including the diurnal rhythm of REM sleep among the various strains of mice [28]. In the present study, the FVB strain of mice have a blunted diurnal rhythm in REM sleep compared to the C57BL/6J strain, but this is consistent with a previously published report [28].

HCRT2-SAP lesions in FVB-GFP mice decreased the percentage of time spent in non-REM sleep (two-way ANOVA, for treatment×day/night, F_1, 28_ = 4.506, P = 0.043) during the day, with no significant difference during the night (fig. 10C and D). Percent wake (two-way ANOVA, for treatment×day/night, F_1, 28_ = 9.943, P = 0.004) in the FVB-GFP lesioned mice decreased during the night and increased in the day (fig. 10E and F). Thus, the *vl*PAG lesion caused the FVB-GFP mice to be awake more during the day.

**Figure 11:**
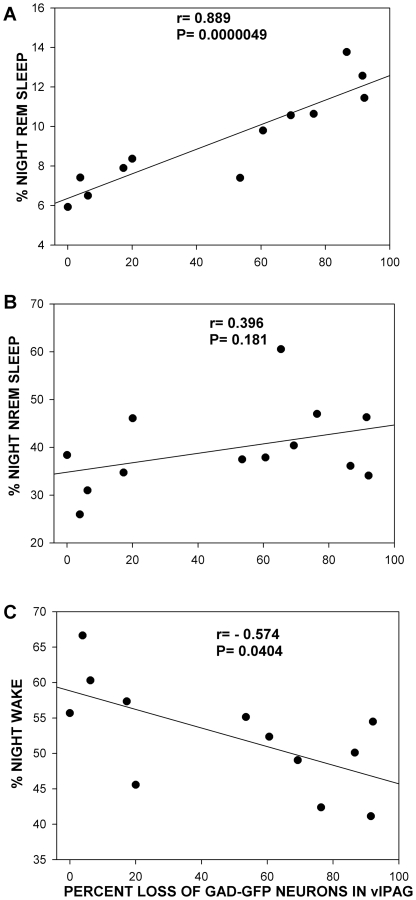
Relationship between loss of GFP neurons in the *vl*PAG and sleep-wake states.

There was a significant correlation between loss of the GFP neurons and REM sleep (r = 0.889; P<0.001) and wake at night (r = −0.574; P = 0.040) in the FVB-GFP lesioned mice (fig. 11). Thus, as more GFP neurons in the *vl*PAG were destroyed the mice were awake less and had more REM sleep at night.

**Figure 12:**
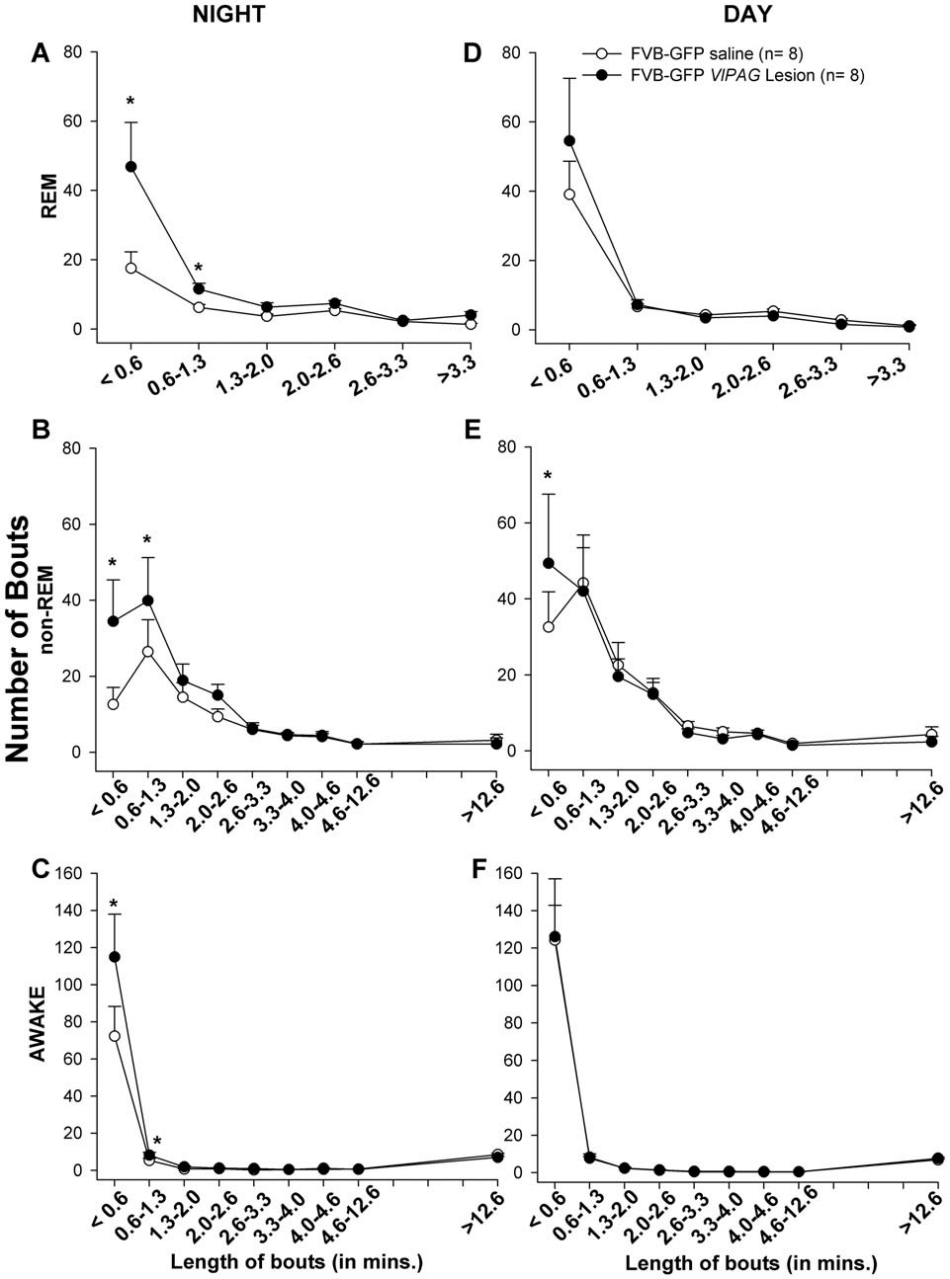
The number and length of bouts of awake, non-REM and REM sleep in saline (open circle) vs. lesioned FVB-GFP mice (closed circle). Each bout of awake, non-REM and REM sleep was placed in a bin of specific length (in minutes) of the bout. The figure represents the average (± SEM) number of bouts of a specific length in the lesioned and saline injected FVB-GFP mice during the dark (A, B, C) and light phase (D, E, F). Asterisk (*) represents p<0.01 lesioned vs. saline in FVB-GFP mice.

As with lesions in the HCRT*-ko* mice *vl*PAG lesions produced a significant increase in the number of REM bouts (F_1, 14_ = 7.762, P = 0.015) and decrease in the length of wake bouts (F_1, 14_ = 6.107, P = 0.027) during the night phase (Table-1). These lesions also increased the number of shorter bouts (<1.3 minutes) of REM, non-REM and wake state predominantly during the dark phase (Fig. 12) and resulted in a trend of 58% increase in number of transitions during the night time, similar to that seen in the HCRT*-ko* mice. However, the effect on the state transitions was not statistically significant.

### Effects on cataplexy

Lesions of the *vl*PAG did not produce cataplexy in the FVB-GFP transgenic mice. *vl*PAG lesions increased the incidence of REM sleep bouts but there was no evidence of cataplexy.

### Experiment 3: Input to the *vl*PAG

**Figure 13:**
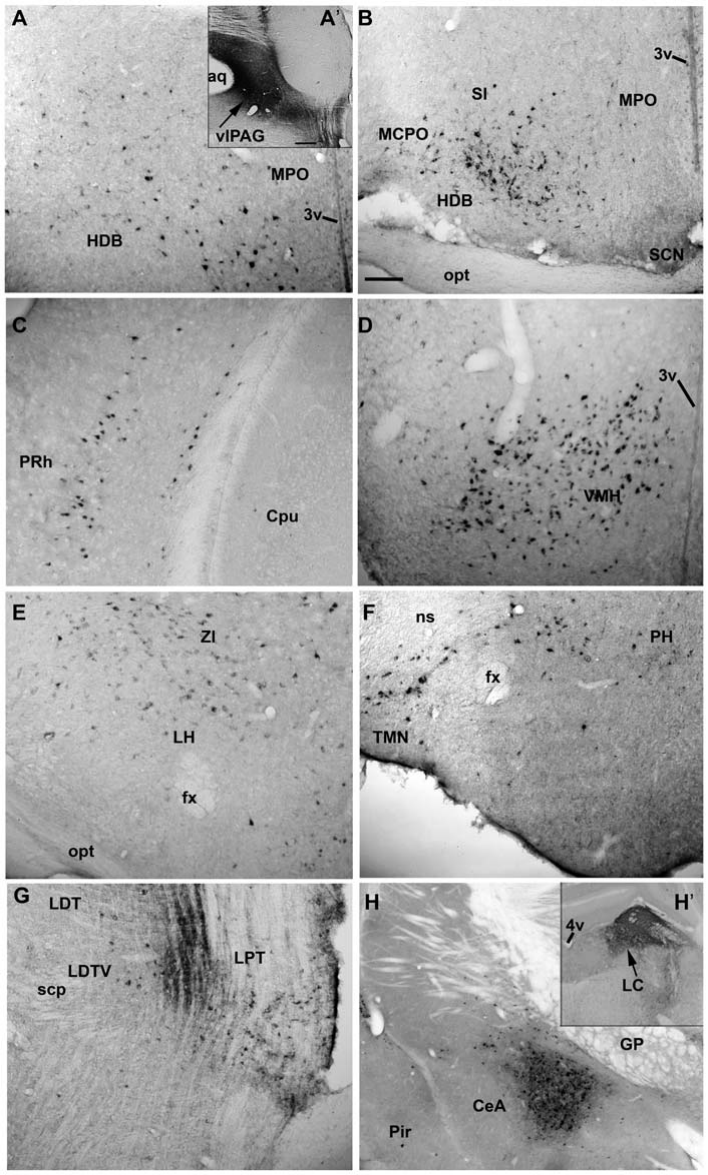
Efferent input to the *vl*PAG area. An injection of 23 nl of 0.5% CTB in *vl*PAG (shown the A') resulted in the retrolabeling of cells in the following areas: A- Basal forebrain (Medial preoptic area): B- Basal forebrain (magnocellular preoptic area); C- Perirhinal cortex; D- ventro-medial hypothamalus; E- lateral hypothalamus; F- tuberomammillary nucleus in the posterior hypothalamus and G- lateral pontine tegmentum. A caudal spread of injection to the locus coeruleus was seen in two cases (shown in H') and it resulted in retrograde labeling of cells in the amygdala as shown in H. Scale in A' and H' = 200 µm; A-H scale = 100 µm.Abbreviations: 3v- third ventricle; 4v- fourth ventricle; aq- aquaduct; CeA- central nucleus of Amygdala; Cpu- caudate putamen; fx- fornix; GP- globus pallidus; HDB- horizontal limb of the diagonal band of broca; LC- locus coeruleus; LDT- dorsolateral tegmentum; LDTV- ventral dorsolateral tegmentum; LH- lateral hypothalamus; LPT- lateral pontine tegmentum; MCPO- magnocellular preoptic area; MPO- medial preoptic; ns- nigrostriatal bundle; opt- optic tract; PH- posterior hypothalamus; Pir- Piriform cortex; PRh- Perirhinal cortex; SCN- suprachiasmatic nucleus; scp- superior cerebellar peduncle; SI- substantia innominata; TMN- tuberomammillary nucleus; *vl*PAG- ventrolateral preoptic area; VMH- ventromedial hypothalamus.

**Figure 14:**
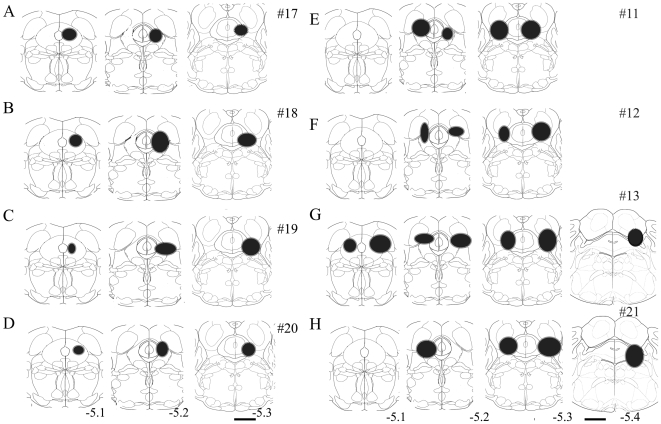
Location and spread of the retrograde tracer, cholera toxin subunit b (CTb) in the vlPAG area, as observed by the immunohistochemistry for the CTb in the eight mice (A-H). In mice # 17–19 the CTb was injected unilaterally (right side), whereas in mice 11, 12, 13 and 21 it was injected bilaterally into the vlPAG area. In one mouse (# 21) the CTb spread caudally into the locus coeruleus as shown in H. The numbers of retrogradelylabeled neurons in specific brain regions in each mouse is summarized in Table 2. Scale = 1 mm.

The retrograde tracer cholera toxin subunit b (CTb) was injected into the *vl*PAG area, either on one side or both sides (Figure 13A' and H'). The site and spread of the CTb is depicted in Figure 14. The numbers of retrogradely labeled neurons were counted on both the ipsilateral and contralateral sides (Table 2, figure 13). Moderate to high numbers of retrogradely filled neurons were found in the central nucleus of the amygdala, magnocellular preoptic nucleus, preoptic area, perirhinal cortex, ventro-medial hypothalamus (VMH), lateral hypothalamus (LH), tuberomammillary nucleus (TMN), and lateral pontine tegmentum (Figure 13A-H). Some of the CTb labeled cells in lateral hypothalamus were immunoreactive for hypocretin (Fig. 7B) and melanin concentrating hormone (MCH), suggesting that both MCH and hypocretin have inputs to the *vl*PAG area. When the CTb was located more caudally and encroached onto the locus coeruleus, the numbers of retrogradely labeled neurons in the amygdala increased (Figure 13, 14 and Table 2).

**Table 2:** Number of reterogradely labeled neurons in different brain areas following injection of CTb into the ventrolateral periaquaductal gray area (vlPAG).

Mouse#	#17 (A)		#18 (B)		#19 (C)		#20 (D)		#11 (E)		#12 (F)		#13 (G)		#21 (H)	
Laterality	L (Contra)	R (Ipsi)	L (Contra)	R (Ipsi)	L (Contra)	R (Ipsi)	L (Contra)	R (Ipsi)	L	R	L	R	L	R	L	R
Perirhinal Cortex	–	351	–	170	–	218	–	131	43	31	178	159	154	178	177	287
Central nucleus of Amygdala	–	16	–	21	–	33	–	20	80	6	–	6	11	51	29	389
Medial preoptic area	4	25	33	59	19	94	8	37	75	47	31	33	86	117	59	66
Magnocellular preoptic area	6	60	33	53	28	136	11	34	83	16	25	14	63	165	53	69
Ventromedial hypothalamus	78	151	83	172	169	386	62	216	198	193	272	299	193	263	450	352
Lateral hypothalamus	14	95	33	94	108	212	7	57	109	98	67	46	122	242	162	181
Tuberomammi-llary area	14	61	10	58	36	170	4	84	81	65	22	16	76	85	159	159
Lateral pontine tegmentum	–	80	–	50	–	60	–	56	33	23	47	32	39	32	49	40

The CTb labeled cells were counted bilaterally in the eight mice, the site and the spread of CTb in these mice is shown in figure 14 (A-H). As shown in fig. 14, mice # 17–20 were injected unilaterally on the right side and the table shows the counts of the labeled cells on ipsilateral and contralateral side of the CTb injection. Mice # 11, 12, 13 and 21 were injected bilaterally in vlPAG. Abbreviations: contra = contralateral to injection site; ipsi = ipsilateral to injection site; L = left; R = right.

## Discussion

The primary finding was that the combined deletion of HCRT and neurons in the *vl*PAG exacerbated the REM sleep and sleep fragmentation compared to deletion of HCRT by itself. However, cataplexy, wakefulness, or non-REM sleep were unaffected. When only the *vl*PAG neurons were lesioned REM sleep increased at night, but cataplexy was not triggered. This suggests that the lesioned neurons in the *vl*PAG inhibit REM sleep but not cataplexy. Since the effects were exacerbated in HCRT-ko mice, the vlPAG neurons receive other excitatory inputs, besides HCRT which serves to inhibit REM sleep.

In the present study HCRT2-SAP was used to lesion *vl*PAG neurons. Previously, we demonstrated that the neurotoxin binds to HCRT receptors [6]. Briefly, Chinese hamster ovary (CHO) cells expressing either HCRT-1 or HCRT-2 receptors were exposed to HCRT2-SAP. As negative control, we used KNRK cells (Kirsten murine sarcoma virus transformed Normal Rat Kidney cells) expressing the substance-P receptor. This control tested binding of the HCRT2-SAP to bind to other peptide receptors. Fluorescent activated cell sorting (FACS) analysis was used and determined that, indeed, HCRT2-SAP bound to the HCRT receptor but not the substance-P receptor. As a positive control we determined that substance-P-saporin bound to its receptor. We have also demonstrated that HCRT2-SAP kills neurons in the tuberomammillary nucleus that contain the HCRT-2 receptor [29], whereas the neurons in the locus coeruleus, which contain the HCRT-1 receptor are relatively resistant [12]. Even in the lateral hypothalamus, some neurons are spared [6]. There is no evidence that HCRT2-SAP is transported retrogradely or anterogradely to kill neurons distal to the target site. Moreover, in the present study we did not find any observable evidence of loss of neurons in areas adjacent to the *vl*PAG such as the LC or DRN. Unconjugated saporin did not produce a degree of loss of neurons relative to the conjugated saporin.

The vlPAG area has been lesioned in the cat [14] and rat [16] but this is the first study to lesion it in mice. Considering that in the present study the lesions produced a similar effect on REM sleep in two separate strains of mice, we suggest that the lesioned pontine area inhibits REM sleep. In the present study, the extent of the lesioned area was based on the boundary of loss of NeuN immunoreactivity and encompassed the vlPAG. The lesioned area in the present study in mice was much smaller compared to the vlPAG lesions in rats [16] indicating that a small pontine area inhibits REM sleep. Another area lateral to the vlPAG called the lateral pontine tegmentum (LPT) by one group [16] and the deep mesencephalic reticular nucleus (DPMe) by another [10] is also implicated in inhibiting REM sleep and cataplexy. In the present study, lesions may have encroached into the LPT, but more complete lesions of this area need to be made in HCRT-ko mice to fully understand its role in REM sleep.

In experiment 1 HCRT*-ko* mice were used. These mice have cataplexy, increased REM sleep at night, and more fragmented sleep architecture compared to WT mice [17]. We reasoned that if the *vl*PAG neurons are important regulators of REM sleep and muscle tone then deletion of both the ligand and the target neurons in the *vl*PAG should have an additive effect on REM sleep and cataplexy. *vl*PAG lesion in the HCRT*-ko* mice increased REM sleep (+39% compared to HCRT*-ko*; +177% compared to WT), number of bouts of REM sleep, and further fragmented sleep architecture. The lesions did not affect cataplexy, non-REM sleep or wakefulness.

Experiment 2 determined the effects in a system where HCRT was present but the neurons in the *vl*PAG were deleted. The effects of deletion of neurons in the *vl*PAG on sleep in mice are unknown. The HCRT*-ko* mice used in experiment 1 have a C57BL/6J background. Rather than lesion the *vl*PAG in C57BL/6J wildtype mice, which we reasoned would only incrementally advance our understanding of the network we chose a different strain of mice, the FVB strain. This strain like the C57BL/6J is also an inbred strain, but it is preferred for transgenic analysis [30]. We chose an FVB strain where eGFP is expressed in GABA neurons [21]. We reasoned that another strain of mice, one where the phenotype of underlying neurons could be more easily identified with reporter genes such as eGFP, would facilitate tract-tracing and pharmacology. For instance, in electrophysiology studies the eGFP neurons could be recorded in a slice preparation to identify the pharmacology of these neurons. Therefore, we selected the FVB strain, since in these transgenics eGFP is driven by a murine Gad-1 gene (one of the Gad isoforms) [21].

First, we verified that virtually all of the eGFP neurons in the *vl*PAG were also GABA positive (Fig. 6), and then determined that these neurons were also immunoreactive for the HCRT-2 receptor antibody. A previous report identified HCRT receptor mRNA in the *vl*PAG of rats [27], but in the present study immunohistochemistry determined that HCRT-2 receptor was present in eGFP/GABA neurons in the mouse *vl*PAG (Figure 7D). The HCRT-receptor 2 antibody that was used has recently been tested to be specific to HCRT-2 receptors [22]. In the present study HCRT-2 receptor immunoreactivity was not found in the locus coeruleus where instead the HCRT-1 receptor is present. These verifications are a necessary first step because now these transgenic mice can be used in electrophysiology or anatomical tract tracing studies to identify the role of the GABA vlPAG neurons in the REM sleep circuit.

The second step was to determine the effects of lesion of the *vl*PAG in the FVB strain of mice. *vl*PAG lesion in the FVB-GFP transgenic mice produced a 79% increase in REM sleep at night, and a 43% increase over the 24 h period compared to non-lesioned transgenic mice. The loss of the GFP neurons in the *vl*PAG was positively correlated (r = 0.889) with REM sleep. The lesioned mice also slept more at night compared to non-lesioned mice. Lesions of the *vl*PAG produced a significant increase in the number of REM sleep bouts and decrease in the length of wake bouts during the night phase (Table-1). No bouts of cataplexy were evident. Thus, use of the GFP transgenic line bolstered the results from experiment 1. More importantly, this study demonstrates the benefits of these transgenic mice in sleep studies.

Experiment 3 was performed to identify inputs to the *vl*PAG. There was a prominent input from the wake active areas such as the ventromedial hypothalamus (VMH), posterior lateral hypothalamus (pLH), lateral hypothalamus (LH), tuberomammillary nucleus (TMN) and lateral pontine tegmentum (LPT). Additionally, neurons from the central nucleus of the amygdala, and perirhinal cortex project to the *vl*PAG.

**Figure 15:**
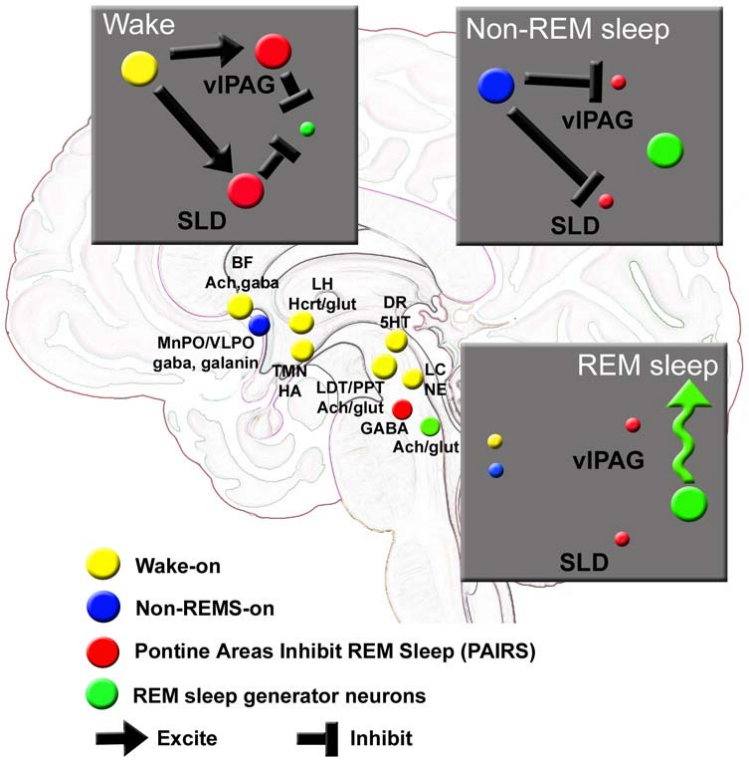
Model of neuronal populations that regulate wake, non-REM sleep and REM sleep (9, 13, 16). There are several neuronal populations that are considered to generate wakefulness and these are identified in yellow. Neurons that are considered to generate non-REM sleep are identified in blue. Both of these neuronal populations act on neurons in the pontine brainstem (red) and influence the generation of rapid-eye movement sleep (REM sleep). Wake-on neurons inhibit REM sleep by activating pontine GABA neurons in the pons. The strength of the excitatory input to the pontine GABA neurons influences REM sleep. A strong input will inhibit REM sleep while a weakened input will facilitate it. During non-REM sleep the excitatory input to the pontine GABA neurons is lost and is replaced by a strong inhibitory input. This enables REM sleep generator neurons (green) to become active and when sufficient numbers of these are activated then REM sleep ensues. In the present paper we tested this model by lesioning the neurons in the ventral lateral periaquaductal gray area (vlPAG), and found that REM sleep was significantly increased. When these neurons were lesioned in the hypocretin knockout mice (HCRT-*ko*) then the REM sleep increase was exacerbated compared to HCRT-ko mice without vlPAG lesion. The SLD has been lesioned in rats (12,16) but not mice and there is an increase in REM sleep supporting the hypothesis of PAIRS. Abbreviations: Ach = acetylcholine; BF = basal forebrain; DR = dorsal raphe; GABA = gamma amino butyric acid; GLUT = glutamic acid; HA = histamine; HCRT = hypocretin; LC = locus coeruleus; LH = lateral hypothalamus; MnPO = median preoptic nucleus; NE = norepinephrine; LDT = lateral dorsal pontine tegmentum; PPT = pedunculopontine tegmentum; SLD = sub lateral dorsal nucleus; TMN = tuberomammillary nucleus; vlPAG = ventral lateral periaquaductal gray; VLPO = ventral lateral preoptic nucleus.

What is the significance of this input to the vlPAG? The perirhinal cortex plays an important role in memory especially with the novelty and familiarity of objects [31], and it interacts with the hippocampus to strengthen memory [32]. The input from the central nucleus of the amygdala would be relevant for emotion [33]. Both of these may help direct the animal to a familiar food source. The inputs from the VMH and LH would arouse the animal because glucosensing neurons in these regions [34] respond to a very narrow range of glucose levels (0.1 to 2.5 mmol/l). A lower glucose level, which is likely at the end of a rodent's sleep period (during the day in nocturnal rodents), would activate arousal neurons so that the animal can forage for food and restore energy balance. Indeed, the HCRT neurons are activated by low glucose levels and shut-off when the glucose levels increase [35]. Taken together, we suggest that the projections from areas associated with memory, emotion, glucosensing and arousal neurons would have an excitatory influence on the *vl*PAG GABA neurons (Figure 15). We suggest that the activation of the *vl*PAG GABA circuit prevents the animal from entering into REM sleep thereby enabling it to maintain the proper posture and vigilance required for foraging and feeding.

The results from experiment 3 indicate that the vlPAG neurons receive input from a number of regions including HCRT neurons. Together these inputs excite the *vl*PAG GABA neurons to inhibit REM sleep. Considering that REM sleep was further increased after vlPAG lesions in HCRT-ko mice is evidence that other non-HCRT input to the *vl*PAG acts to inhibit REM sleep. The neurotoxin may have also destroyed non-HCRT receptor bearing neurons, and this may also have had an additive effect.

An important finding of this study was that lesions of the *vl*PAG area increased REM sleep but not cataplexy. The HCRT-2 receptor is widely present on the *vl*PAG neurons, including the eGFP/GABA neurons and all of these neurons were likely lesioned by the neurotoxin, but only REM sleep increased. Cataplexy is a state that is distinct from REM sleep both at the behavioral and electrophysiological level [25], and therefore the neural circuit that triggers it is also likely to be separate. Since cataplexy was not affected it is possible that neurons regulating muscle tone were not lesioned (since they do not contain the HCRT receptor) or that the cataplexy neurons are not located in the *vl*PAG. The second possibility is likely since in a recent study we directly monitored the electrophysiological activity of *vl*PAG neurons and did not find any cataplexy-on neurons in the *vl*PAG of HCRT*-ko* mice [25].

What is the activity of *vl*PAG neurons during wake, non-REM sleep, REM sleep and cataplexy? This question has now been answered by our group in a study that directly monitored the electrophysiological activity of *vl*PAG neurons during sleep-wake states and cataplexy [25]. We monitored the activity of *vl*PAG neurons in HCRT*-ko* mice and found that 64% of neurons sampled (28 of 44) were active during both wake and REM sleep, 27% (12 of 44) were wake-active, and 9% (4 of 44) were active during REM sleep [25]. No neurons were active during cataplexy. Such a study has not been done in canine narcolepsy where neuronal activity during cataplexy can also be recorded. The range of activity across sleep-wake states is consistent with data in the cat [36]. Thus, on the basis of electrophysiology most (40/44) *vl*PAG neurons are either wake-active or wake-REM active. Since they are active during wake they are likely driven by HCRT, possess the HCRT receptor and lesioned in the present study. Particularly interesting and relevant to this study was that a subset (9%) of *vl*PAG neurons were more active during REM sleep compared to waking, non-REM sleep or cataplexy. We suggest that these neurons may be key to REM sleep generation, but not cataplexy and their activity may underlie the increased REM sleep in the present study. Juxtacellular recording of *vl*PAG neurons in the FVB-GFP mice could elucidate the circuit. Moreover, it is important to determine projections of these GFP-GABA neurons.

This is the first study to find an additive effect of deletion of both HCRT and HCRT receptor bearing neurons in the pons. These results underscore the importance of *vl*PAG neurons in regulating REM sleep. Upon discovery of the link between HCRT and REM sleep, initial models emphasized the role of the locus coeruleus (LC) and the histaminergic neurons of the tuberomammillary nucleus (TMN) in controlling REM sleep. Both the LC and the TMN receive among the heaviest projections of HCRT neurons. However, specific lesions of both these targets, either single lesion or in combination (triple), do not increase REM sleep [37]. Indeed, even in mice lacking both HCRT and the norepinephrine synthesizing enzyme, dopamine beta hydroxylase, i.e., a double knockout, there is no additive effect [38].

The vlPAG is one pontine area that inhibits REM sleep (see Figure 15). Another area is located in a region ventral to the locus coeruleus and also ventral to the sub-lateral dorsal region (SLD) [13]. Neurons in this area also inhibit REM sleep because when we lesioned it using HCRT2-SAP there was a significant increase in REM sleep [12]. Subsequently, other investigators found that non-specific lesions of this region with ibotenic acid also increase REM sleep in rats [16]. Thus, multiple pontine areas inhibit REM sleep (Fig. 15). Because lesions of these areas and/or a hypocretin deficiency typically increase REM sleep at night, it will be important to determine the effects such lesions in diurnal rodents.

## Supporting Information

Figure S1 Effect of unconjugated saporin (SAP) on vlPAG neurons in a representative wildtype C57BL/6J mouse. Photo A depicts abundant NeuN labeled neurons surrounding the SAP microinjection site in the vlPAG. To verify that SAP was injected green fluorescent beads (2%) were added and photo B shows dispersal of beads at tip of injection site (photo B). Photo C is of a bead adjacent to the perikarya of a hypocretin-2 receptor bearing neuron (white arrow) indicating the ineffectiveness of unconjugated SAP to kill neurons relative to the conjugated version. Photos A and B are of the same tissue section, and Photo C is from an adjacent tissue section that was processed for visualization of the HCRT-2 receptor.
(4.99 MB TIF)
