# Supplementary figures and images for: Hypocretin-2 Saporin Lesions of the Ventrolateral Periaquaductal Gray (vlPAG) Increase REM Sleep in Hypocretin Knockout Mice
Source: PLoS One. 2009 Jul 22;4(7):e6346. doi: 10.1371/journal.pone.0006346 (PMC2709920; doi:10.1371/journal.pone.0006346)

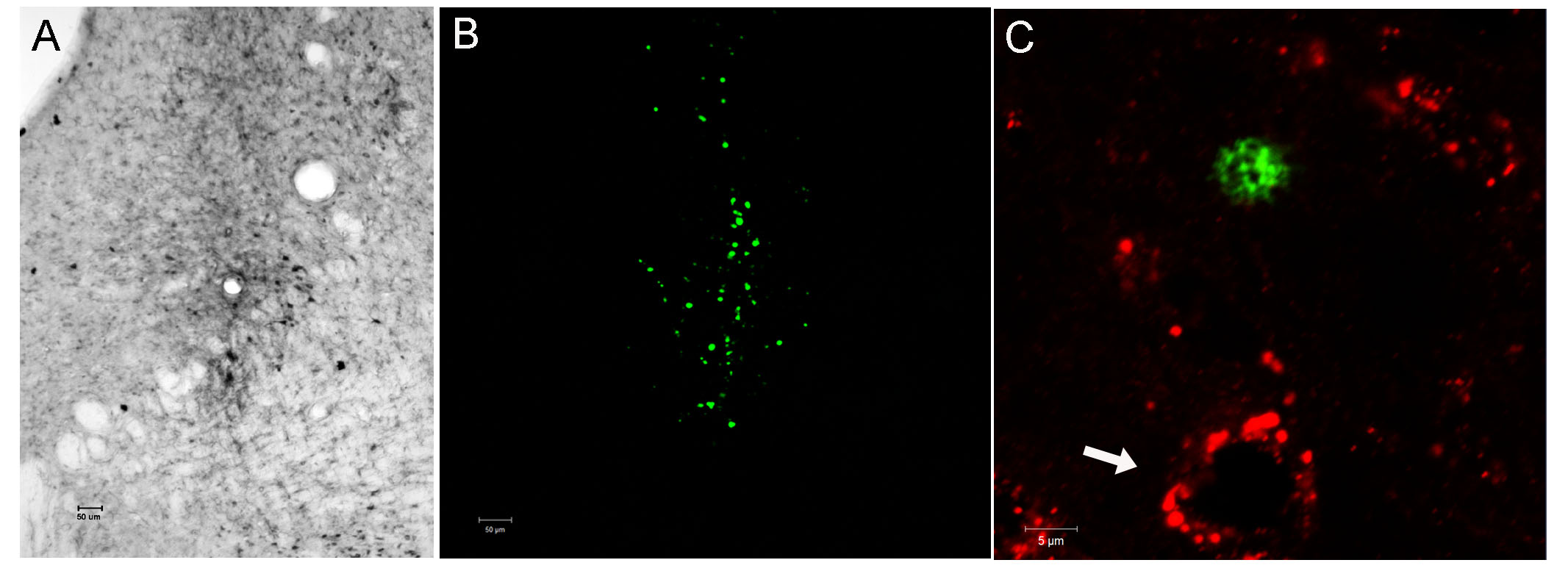

Supplement: Figure S1 — Effect of unconjugated saporin (SAP) on vlPAG neurons in a representative wildtype C57BL/6J mouse. Photo A depicts abundant NeuN labeled neurons surrounding the SAP microinjection site in the vlPAG. To verify that SAP was injected green fluorescent beads (2%) were added and photo B shows dispersal of beads at tip of injection site (photo B). Photo C is of a bead adjacent to the perikarya of a hypocretin-2 receptor bearing neuron (white arrow) indicating the ineffectiveness of unconjugated SAP to kill neurons relative to the conjugated version. Photos A and B are of the same tissue section, and Photo C is from an adjacent tissue section that was processed for visualization of the HCRT-2 receptor. (4.99 MB TIF) [file pone.0006346.s001.tif]
